# Supplementary material for: Plasmids promote antimicrobial resistance through Insertion Sequence-mediated gene inactivation
Source: Nat Microbiol. Author manuscript; Available in PMC 2026 Oct 30. (PMC7619650; doi:10.1038/s41564-026-02290-x)
Supplement: Supplementary file 1 [file EMS216053-supplement-Extended_Data_Fig__1.pdf]

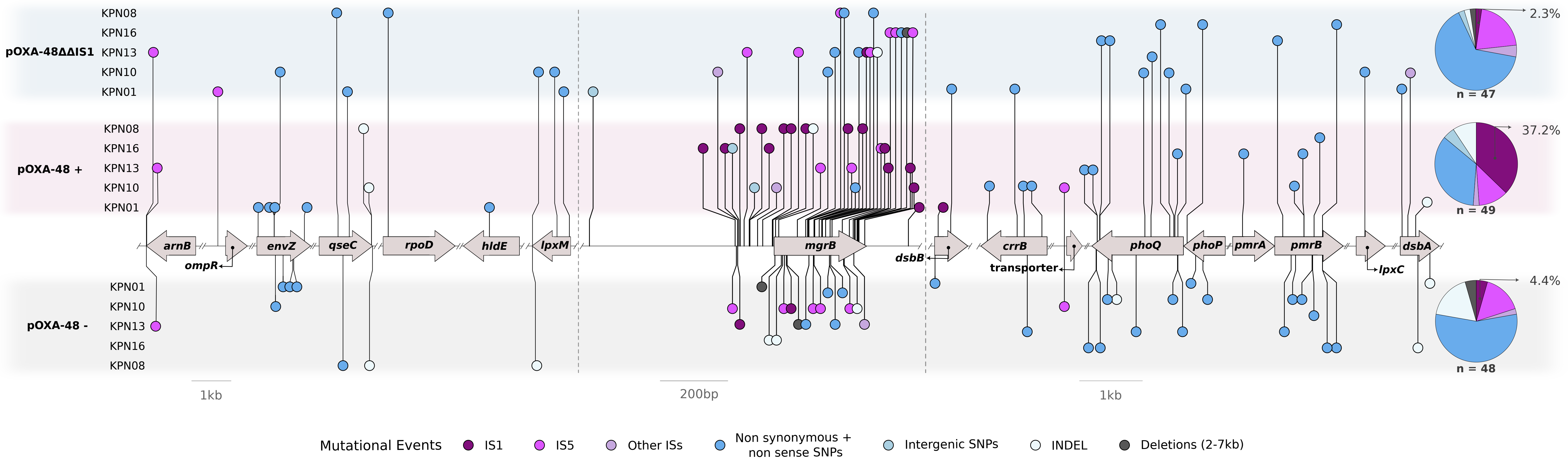

pOXA-48ΔΔIS1
pOXA-48 +
pOXA-48 -
KPN08
KPN16
KPN13
KPN10
KPN01
arnB
ompR
envZ
qseC
rpoD
hldE
lpxM
mgrB
dsbB
crrB
transporter
phoQ
phoP
pmrA
pmrB
lpxC
dsbA
2.3%
n = 47
37.2%
n = 49
4.4%
n = 48
1kb
200bp
1kb
Mutational Events
IS1
IS5
Other ISs
Non synonymous + non sense SNPs
Intergenic SNPs
INDEL
Deletions (2-7kb)
