# Supplementary material for: Plasmids promote antimicrobial resistance through Insertion Sequence-mediated gene inactivation
Source: Nat Microbiol. Author manuscript; Available in PMC 2026 Oct 30. (PMC7619650; doi:10.1038/s41564-026-02290-x)
Supplement: Supplementary file 2 [file EMS216053-supplement-Extended_Data_Fig__10.pdf]

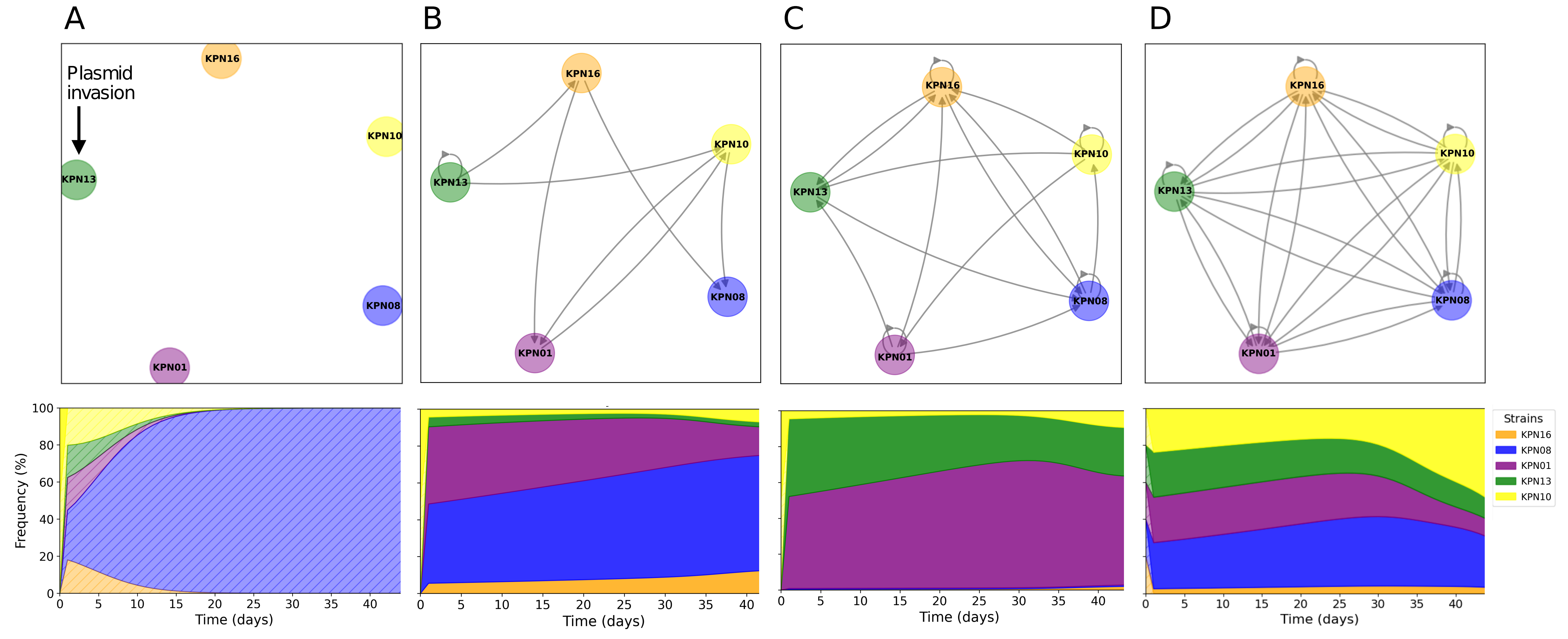
A
B
C
D
Plasmid invasion
KPN16
KPN10
KPN13
KPN08
KPN01
Frequency (%)
Time (days)
Strains
KPN16
KPN08
KPN01
KPN13
KPN10
