# Supplementary material for: Plasmids promote antimicrobial resistance through Insertion Sequence-mediated gene inactivation
Source: Nat Microbiol. Author manuscript; Available in PMC 2026 Oct 30. (PMC7619650; doi:10.1038/s41564-026-02290-x)
Supplement: Supplementary file 3 [file EMS216053-supplement-Extended_Data_Fig__2.pdf]

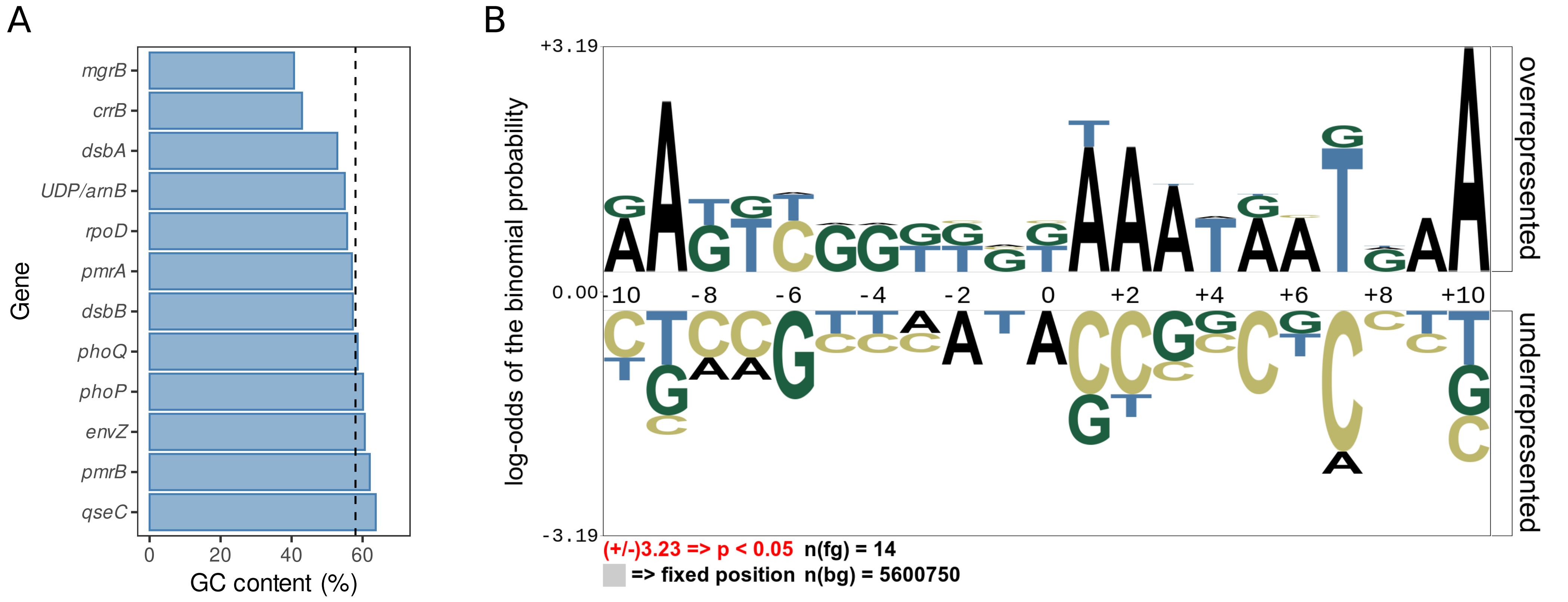

A
mgrB
crrB
dsbA
UDP/arnB
rpoD
pmrA
dsbB
phoQ
phoP
envZ
pmrB
qseC
Gene
0
20
40
60
GC content (%)
B
+3.19
0.00
-3.19
log-odds of the binomial probability
-10
-8
-6
-4
-2
0
+2
+4
+6
+8
+10
overrepresented
underrepresented
(+/-)3.23 => p < 0.05
n(fg) = 14
=> fixed position
n(bg) = 5600750
