# Supplementary material for: Plasmids promote antimicrobial resistance through Insertion Sequence-mediated gene inactivation
Source: Nat Microbiol. Author manuscript; Available in PMC 2026 Oct 30. (PMC7619650; doi:10.1038/s41564-026-02290-x)
Supplement: Supplementary file 4 [file EMS216053-supplement-Extended_Data_Fig__3.pdf]

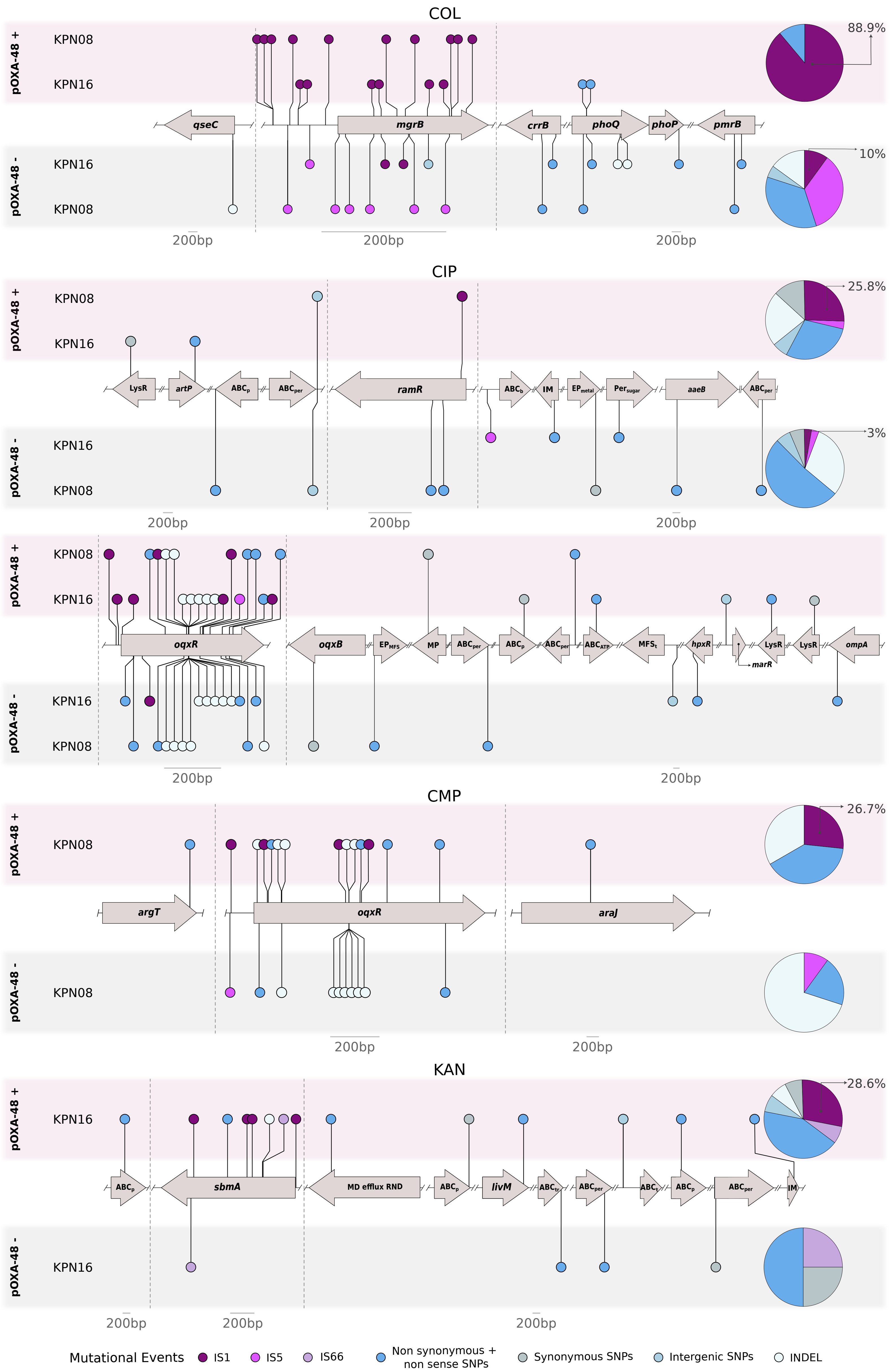

COL
pOXA-48 +
KPN08
KPN16
pOXA-48 -
KPN16
KPN08
qseC
mgrB
crrB
phoQ
phoP
pmrB
88.9%
10%
200bp
200bp
200bp
CIP
LysR
artP
ABCp
ABCper
ramR
ABCb
IM
EPmetal
Persugar
aaeB
ABCper
25.8%
3%
oqxR
oqxB
EPMFS
MP
ABCper
ABCp
ABCper
ABCATP
MFSt
hpxR
marR
LysR
LysR
ompA
CMP
argT
oqxR
araJ
26.7%
KAN
ABCp
sbmA
MD efflux RND
ABCp
livM
ABCtr
ABCper
ABCt
ABCp
ABCper
IM
28.6%
Mutational Events
IS1
IS5
IS66
Non synonymous + non sense SNPs
Synonymous SNPs
Intergenic SNPs
INDEL
