# Supplementary material for: Plasmids promote antimicrobial resistance through Insertion Sequence-mediated gene inactivation
Source: Nat Microbiol. Author manuscript; Available in PMC 2026 Oct 30. (PMC7619650; doi:10.1038/s41564-026-02290-x)
Supplement: Supplementary file 5 [file EMS216053-supplement-Extended_Data_Fig__4.pdf]

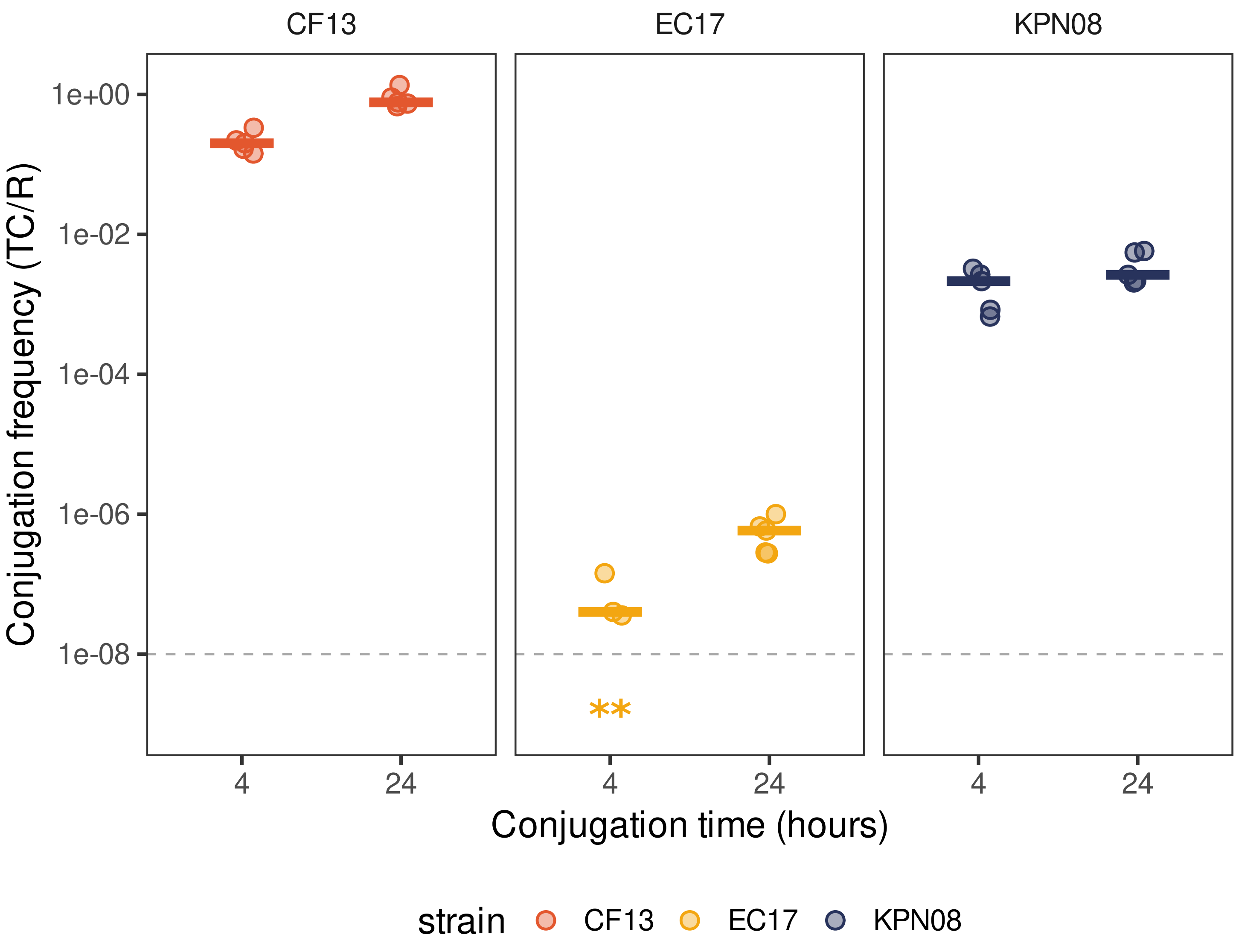

CF13
EC17
KPN08
Conjugation frequency (TC/R)
1e+00
1e-02
1e-04
1e-06
1e-08
**
4
24
4
24
4
24
Conjugation time (hours)
strain
CF13
EC17
KPN08
