# Supplementary material for: Plasmids promote antimicrobial resistance through Insertion Sequence-mediated gene inactivation
Source: Nat Microbiol. Author manuscript; Available in PMC 2026 Oct 30. (PMC7619650; doi:10.1038/s41564-026-02290-x)
Supplement: Supplementary file 6 [file EMS216053-supplement-Extended_Data_Fig__5.pdf]

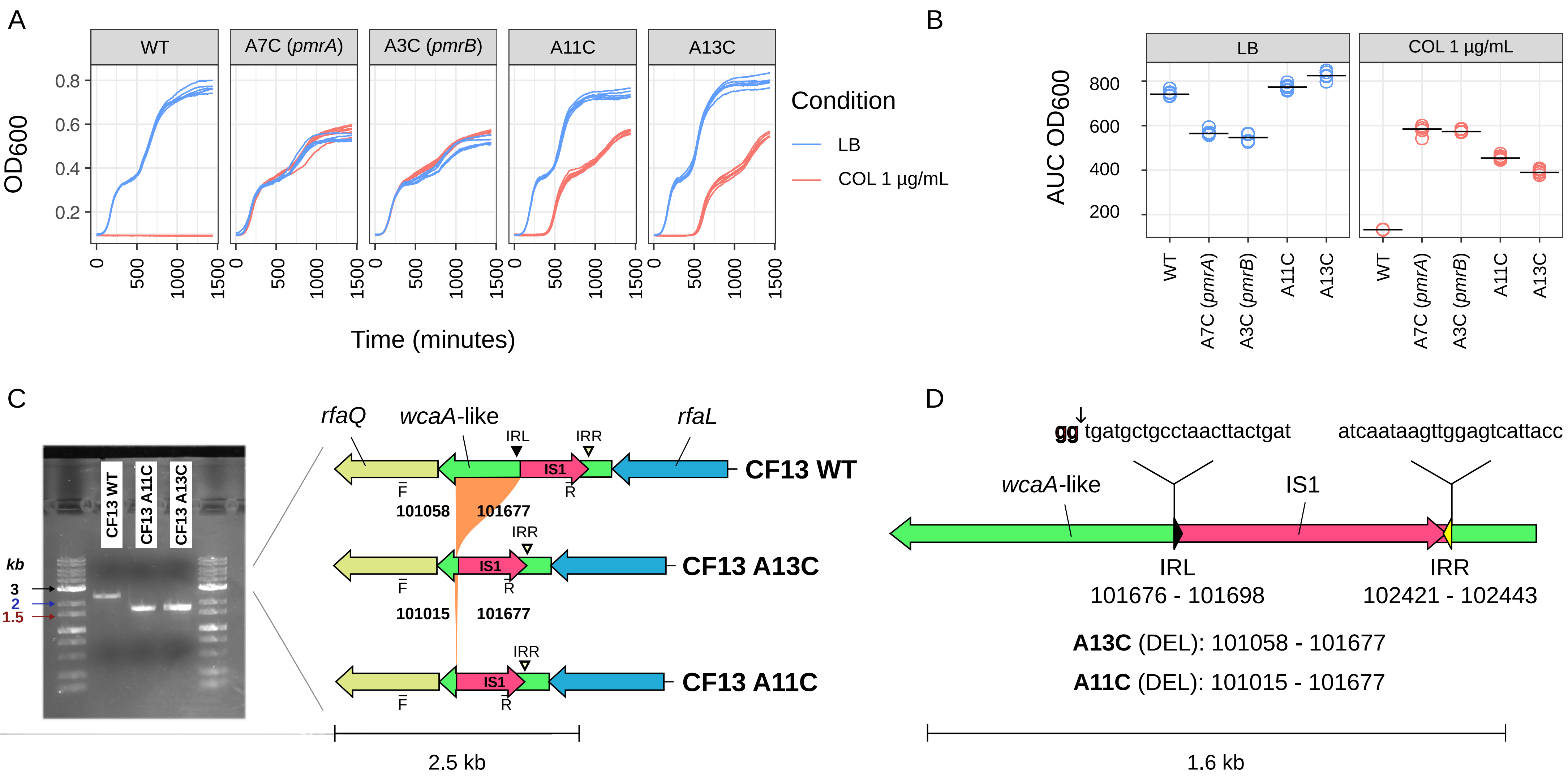
A
WT
A7C (pmrA)
A3C (pmrB)
A11C
A13C
OD600
0.2
0.4
0.6
0.8
0
500
1000
1500
Time (minutes)
Condition
LB
COL 1 µg/mL
B
LB
COL 1 µg/mL
AUC OD600
200
400
600
800
WT
A7C (pmrA)
A3C (pmrB)
A11C
A13C
C
kb
3
2
1.5
CF13 WT
CF13 A11C
CF13 A13C
rfaQ
wcaA-like
rfaL
IRL
IRR
IS1
F
R
101058
101677
101015
CF13 WT
CF13 A13C
CF13 A11C
2.5 kb
D
gg tgatgctgcctaacttactgat
atcaataagttggagtcattacc
wcaA-like
IS1
IRL
101676 - 101698
IRR
102421 - 102443
A13C (DEL): 101058 - 101677
A11C (DEL): 101015 - 101677
1.6 kb
