# Supplementary material for: Plasmids promote antimicrobial resistance through Insertion Sequence-mediated gene inactivation
Source: Nat Microbiol. Author manuscript; Available in PMC 2026 Oct 30. (PMC7619650; doi:10.1038/s41564-026-02290-x)
Supplement: Supplementary file 7 [file EMS216053-supplement-Extended_Data_Fig__6.pdf]

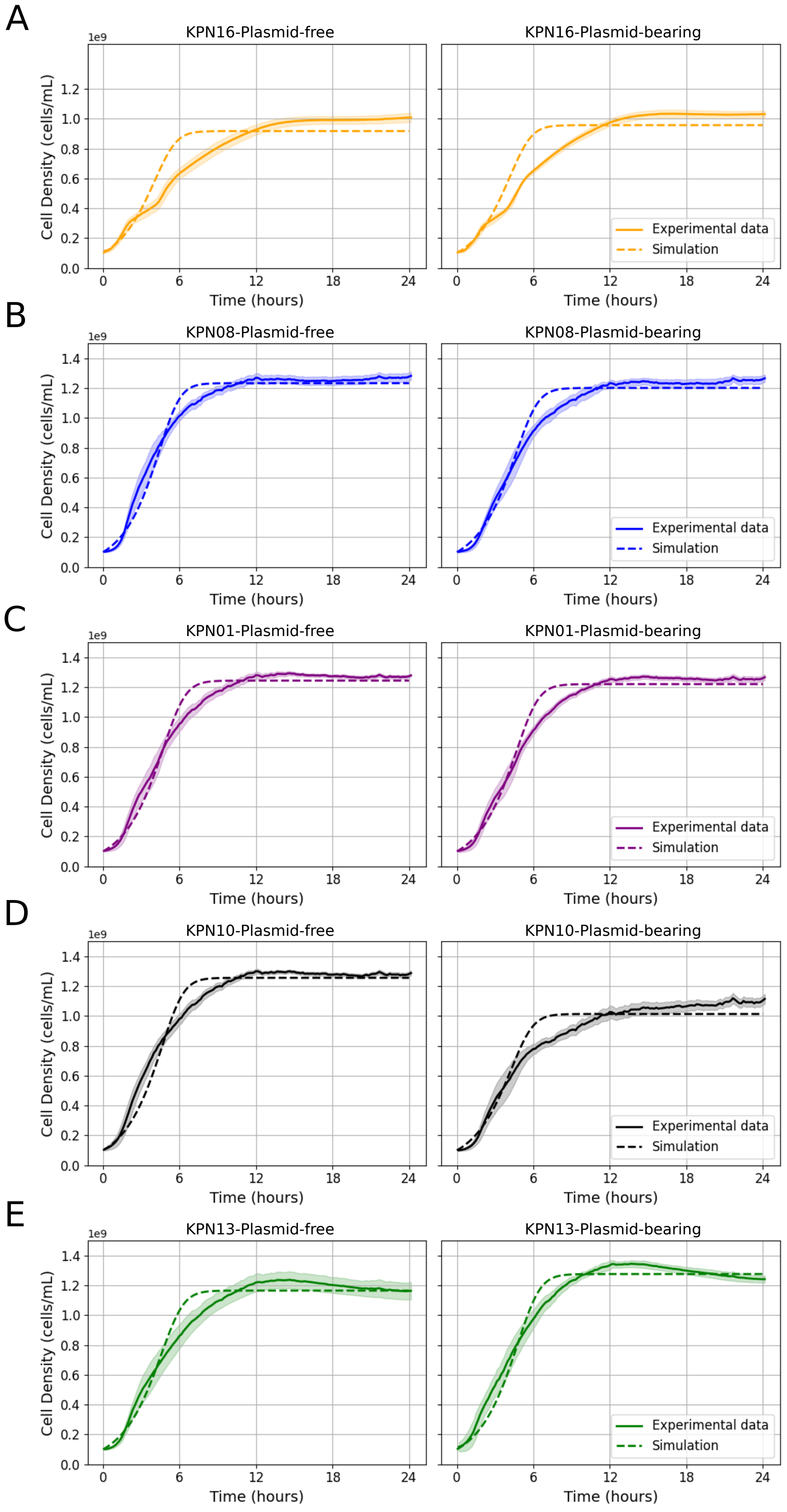
A
KPN16-Plasmid-free
KPN16-Plasmid-bearing
B
KPN08-Plasmid-free
KPN08-Plasmid-bearing
C
KPN01-Plasmid-free
KPN01-Plasmid-bearing
D
KPN10-Plasmid-free
KPN10-Plasmid-bearing
E
KPN13-Plasmid-free
KPN13-Plasmid-bearing
1e9
Cell Density (cells/mL)
Time (hours)
Experimental data
Simulation
