# Supplementary material for: Plasmids promote antimicrobial resistance through Insertion Sequence-mediated gene inactivation
Source: Nat Microbiol. Author manuscript; Available in PMC 2026 Oct 30. (PMC7619650; doi:10.1038/s41564-026-02290-x)
Supplement: Supplementary file 8 [file EMS216053-supplement-Extended_Data_Fig__7.pdf]

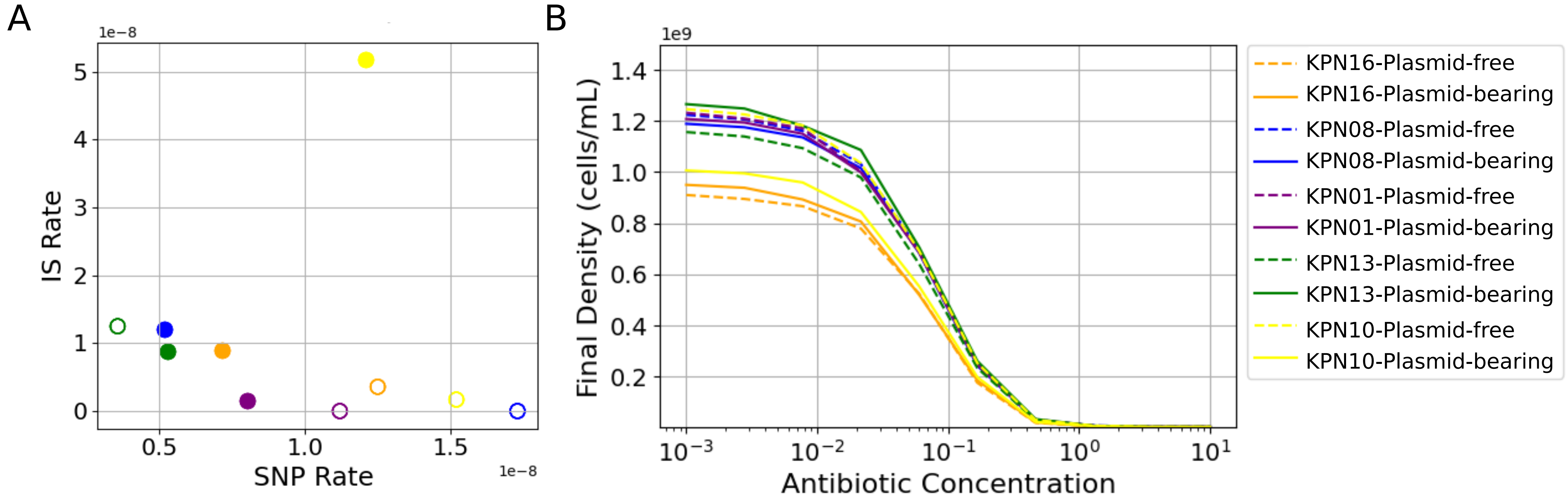

A
1e−8
IS Rate
5
4
3
2
1
0
0.5
1.0
1.5
SNP Rate
1e−8
B
1e9
Final Density (cells/mL)
1.4
1.2
1.0
0.8
0.6
0.4
0.2
10^{-3}
10^{-2}
10^{-1}
10^{0}
10^{1}
Antibiotic Concentration
KPN16-Plasmid-free
KPN16-Plasmid-bearing
KPN08-Plasmid-free
KPN08-Plasmid-bearing
KPN01-Plasmid-free
KPN01-Plasmid-bearing
KPN13-Plasmid-free
KPN13-Plasmid-bearing
KPN10-Plasmid-free
KPN10-Plasmid-bearing
