# Supplementary material for: Plasmids promote antimicrobial resistance through Insertion Sequence-mediated gene inactivation
Source: Nat Microbiol. Author manuscript; Available in PMC 2026 Oct 30. (PMC7619650; doi:10.1038/s41564-026-02290-x)
Supplement: Supplementary file 9 [file EMS216053-supplement-Extended_Data_Fig__8.pdf]

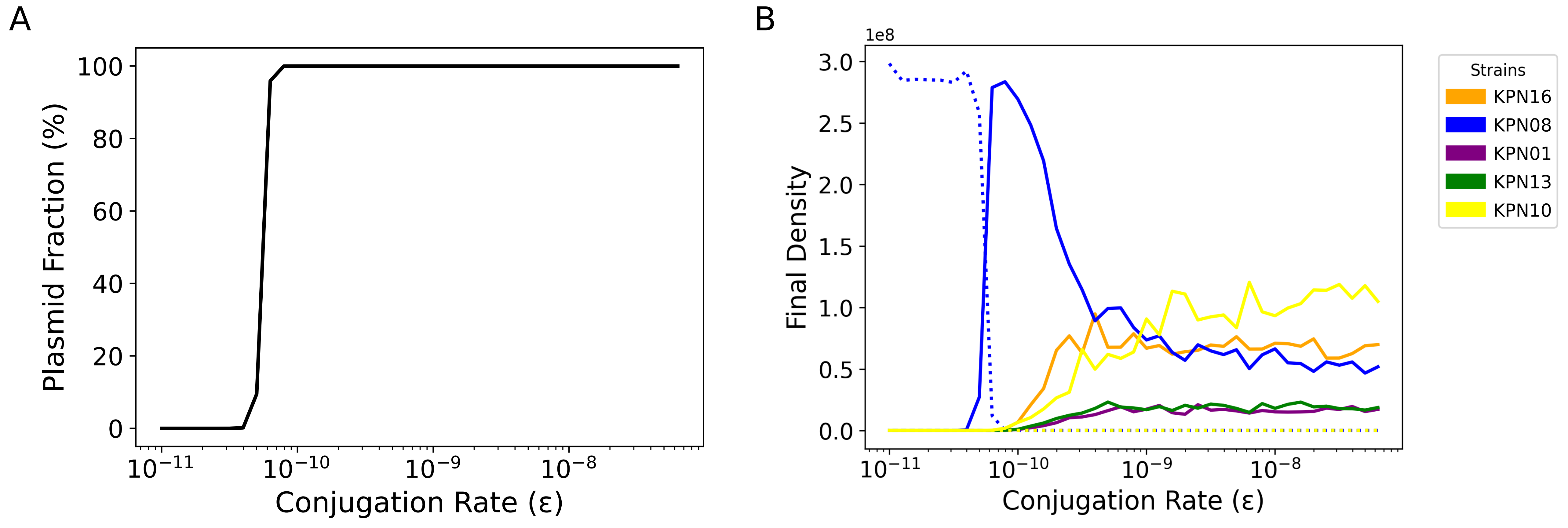
A
Plasmid Fraction (%)
0
20
40
60
80
100
10^{-11}
10^{-10}
10^{-9}
10^{-8}
Conjugation Rate (ε)
B
1e8
Final Density
0.0
0.5
1.0
1.5
2.0
2.5
3.0
10^{-11}
10^{-10}
10^{-9}
10^{-8}
Conjugation Rate (ε)
Strains
KPN16
KPN08
KPN01
KPN13
KPN10
