# Supplementary material for: Plasmids promote antimicrobial resistance through Insertion Sequence-mediated gene inactivation
Source: Nat Microbiol. Author manuscript; Available in PMC 2026 Oct 30. (PMC7619650; doi:10.1038/s41564-026-02290-x)
Supplement: Supplementary file 10 [file EMS216053-supplement-Extended_Data_Fig__9.pdf]

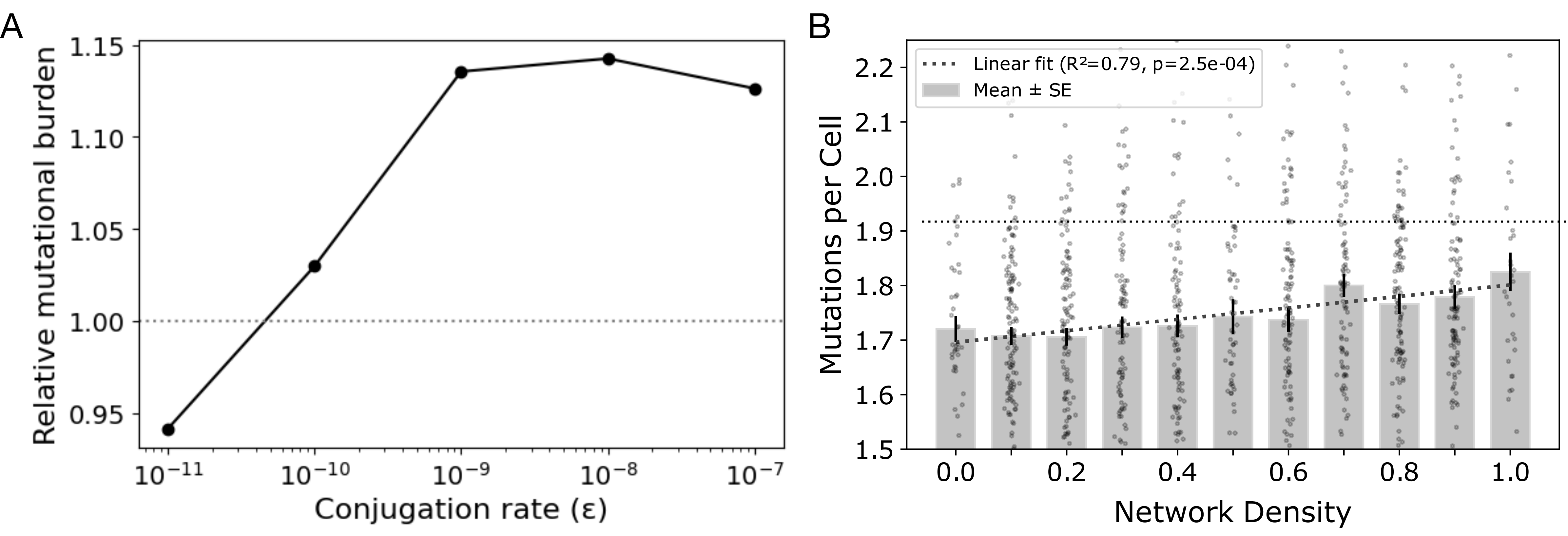

A
Relative mutational burden
0.95
1.00
1.05
1.10
1.15
10^{-11}
10^{-10}
10^{-9}
10^{-8}
10^{-7}
Conjugation rate (ε)
B
Linear fit (R²=0.79, p=2.5e-04)
Mean ± SE
Mutations per Cell
1.5
1.6
1.7
1.8
1.9
2.0
2.1
2.2
0.0
0.2
0.4
0.6
0.8
1.0
Network Density
