# Supplementary material for: Plasmids promote antimicrobial resistance through Insertion Sequence-mediated gene inactivation
Source: Nat Microbiol. Author manuscript; Available in PMC 2026 Oct 30. (PMC7619650; doi:10.1038/s41564-026-02290-x)
Supplement: Supplementary file 11 [file EMS216053-supplement-Supplementary_Information.pdf]

Supplementary Information

Plasmids promote antimicrobial resistance through Insertion Sequence-mediated gene inactivation

Jorge Sastre-Dominguez [1,2*], Paloma Rodera-Fernandez [1,2*], Javier DelaFuente [1], Sandra Martínez-González [1], Susana Quesada [1], Marina Valencoso-Requena [1], Alicia Calvo-Villamañan [1], Coloma Costas [1], Ayari Fuentes-Hernández [3], Alfonso Santos-Lopez [2,4 #], Alvaro San Millan [1,4 #]

[1] Centro Nacional de Biotecnología, Consejo Superior de Investigaciones Cientificas (CSIC), Madrid, Spain.
[2] Departamento de Biología Molecular, Facultad de Biología, Universidad Autónoma de Madrid, Madrid, Spain.
[3] Center for Genomic Sciences, Universidad Nacional Autónoma de México, 62210 Cuernavaca, México.
[4] Consorcio de Investigación Biomédica en Red de Epidemiología y Salud Pública (CIBERESP), Madrid, Spain.
* These authors contributed equally and should be considered as first authors.
[#] Corresponding authors: A.S.-L.: alfonso.santosl@uam.es, A.S.M.: asanmillan@cnb.csic.es

Supplementary Tables

Supplementary tables 1-5 are supplied as independent spreadsheets.

Supplementary Table 6.- Primers used to amplify the marker genes from the community experiment and the wcaA-like gene from CF13.

Name	Sequence (5'->3')	Use
JDF_BckBone-pBGHB-F	gctagttaacTTTTTTTAAGGCAGTTATTGG	Constructing pBGHB
JDF_BckBone-pBGHB-R	cacagggtacTTTAGCTTCCTTAGCTCC	Constructing pBGHB
JDF_HB-F	ggaagctaaaGTACCCTGTGAATAGAGG	Constructing pBGHB
JDF_HB-R	cttaaaaaaaGTTAACTAGCGTACGATC	Constructing pBGHB
JDF_BckBone-pBGZ-F	tgaatgcatcGCCTAGAAATATTTTATCTGATTAATAAG	Constructing pBGZ
JDF_BckBone-pBGZ-F	ttggtcacgaGGGTTATTGTCTCATGAG	Constructing pBGZ
JDF_Zeo-F	acaataacccTCGTGACCAACAGCAACG	Constructing pBGZ
JDF_Zeo-R	atttctaggcGATGCATTCAGTCCTGCTC	Constructing pBGZ
JDF_GFP-F	TTCTGTCAGTGGAGAGGGT	Detecting all pBGC variants (targeting GFP)
JDF_GFP-R	CTGCTAGTTGAACGCTTCCA	Detecting all pBGC variants (targeting GFP)
AC213_LR_IS1oxa_A_P3	agggtaatagagcggccattgaag	Detecting pOXA wt and pOXA-48ΔIS1::kan::cm
AC214_LR_IS1oxa_A&B_P4	cttctcaacccggtacgcac	Detecting pOXA wt and pOXA-48ΔIS1::kan::cm
AC216_LR_IS1oxa_B_P3	gaacaaaacgcgggccaaa	Detecting pOXA wt and pOXA-48ΔIS1::kan::cm
JDF_2278-F	GGATAACGAGCCCGAATGCT	Amplifying the wcaA-like operon in Citrobacter freundii clones
JDF_2278-R	GCAACAGGTCGGTATTGAAGC	Amplifying the wcaA-like operon in Citrobacter freundii clones
JDF_928bpCF13-F	gctggcaataacaggtgtag	Confirming wcaA-like gene deletions in C.freundii clones
JDF_928bpCF13-R	cactggagcacctcaaaaac	Confirming wcaA-like gene deletions in C.freundii clones

Supplementary Table 7.- Parameter values used in the simulations of the computational model.

Parameter	Meaning / where set	Units	Value
M	Number of species	–	5
T	Simulation time	h	24
Δt	Recording interval (dt)	h	0.1
A	Antibiotic concentration	IC90 units	0.0, 0.1, 0.2
σ	Plasmid segregation rate	h^{-1}	1e-07
γ	Conjugation rate sweep	$cell^{-1}h^{-1}$	1e-11…1e-07
c	Resource consumption per division	res. Units · $cell^{-1}$	from fit
μ_{max}	Birth/growth rate	h^{-1}	from fit (matrix)
K_s	Half-saturation (resource)	res. Units	from fit (matrix)
K_a	Half-saturation (antibiotic)	IC90	from fit (0/p matrices)
μ_m	SNP mutation rate	h^{-1}	from fit (matrix)
τ	IS transposition rate	h^{-1}	from fit (matrix)

Supplementary Figures

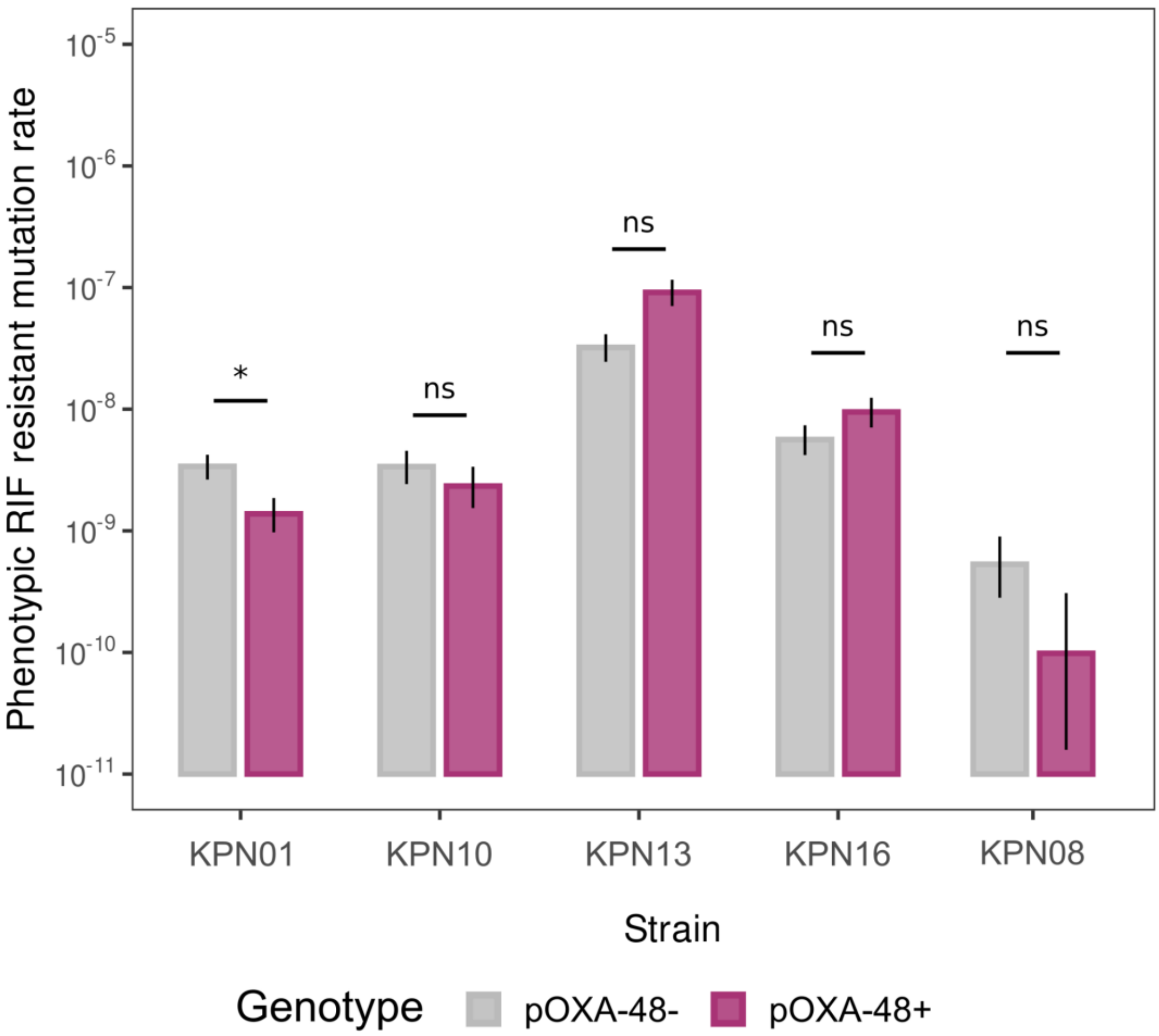

Supplementary Figure 1.- Mutation rate to rifampicin of isogenic clinical pairs carrying or not carrying pOXA-48. Phenotypic resistance mutation rate to rifampicin of the isogenic pairs of *K. pneumoniae* clinical strains either not carrying (grey) or carrying pOXA-48 (purple). The black bars indicate the 95% confidence interval. Asterisks denote statistical significance in the mutation rate between pOXA-48- and pOXA-48+ for each pair based on maximum likelihood ratio tests (n = 35) after Bonferroni-Holm adjustment for multiple testing (p: 0 < *** < 0.001 < ** < 0.01 < * < 0.05 < ns; KPN01 p = 2.7e-02).

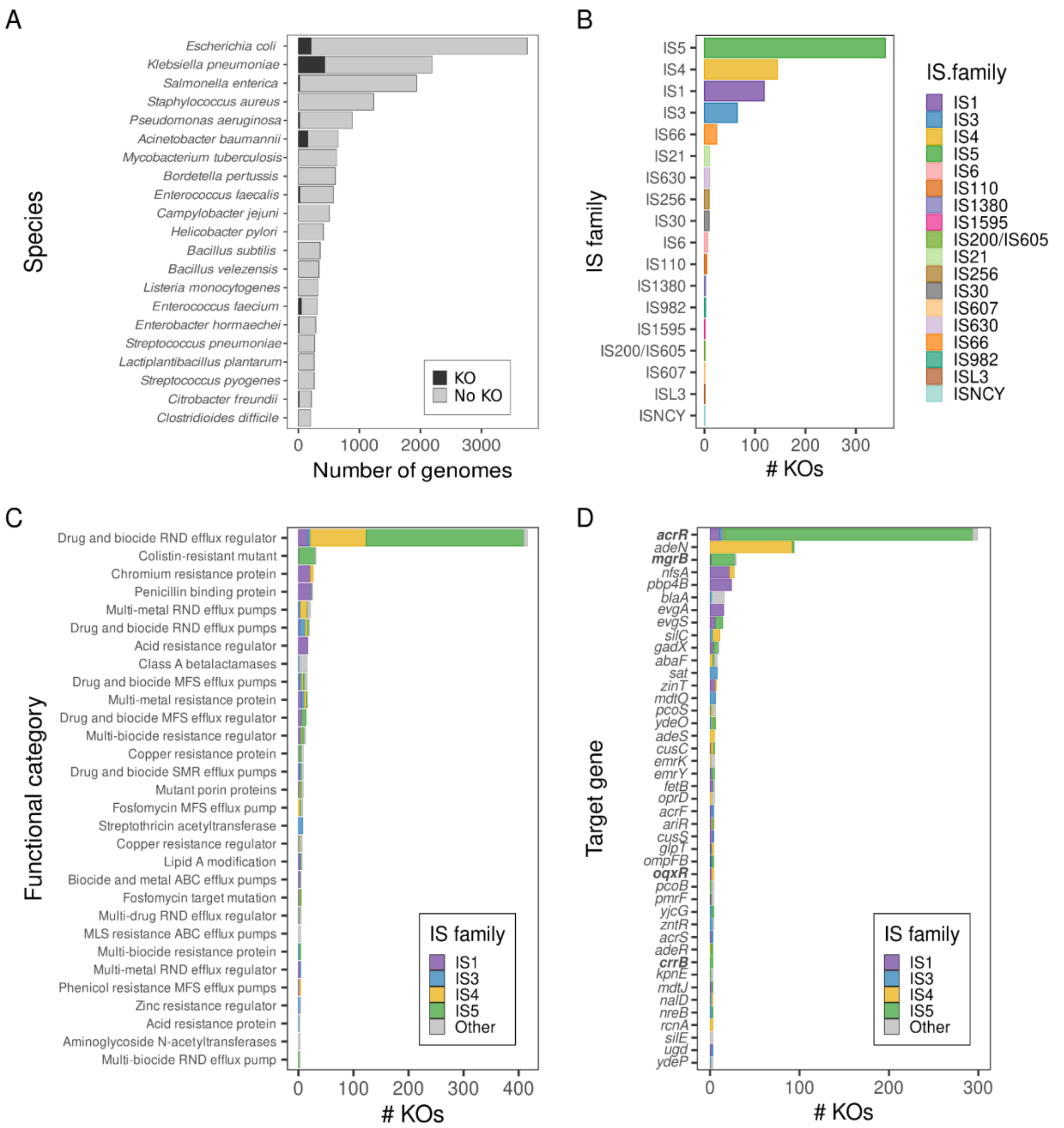

Supplementary Figure 2.- Distribution of IS-mediated KOs in the BV-BRC database. A) Top 20 species analyzed from the BV-BRC database. Darker bars indicate those genomes which showed KO in genes from the MEGARES database and our experimental targets. IS-mediated disruptions were especially prevalent in *K. pneumoniae*, *E. coli* and *A. baumannii*, which are species of high clinical relevance. **B)** Main IS families detected disrupting chromosomal genes from MEGARES+experimental targets. IS5, IS4, IS1 and IS3 families were the main families driving KO-AMR. **C)** Main functional categories targeted by IS elements in the ~42K genomes analyzed from BV-BRC. We observed that most of the targets corresponded to RND efflux pump regulators, followed by colistin resistance related genes. **D)** Main gene targets disrupted by IS elements. Most insertions inactivated the efflux pump regulators *acrR* and *adeN*, which disruption confers MDR phenotypes in Enterobacterales and *A. baumannii* respectively. As expected, we also detected disruptions in *mgrB*. Other genes, such as *nfsA*, which inactivation confers resistance to nitrofurantoin, were among the top detected targets. Bold gene names indicate those genes which were also targeted in our experiments. Main IS families are colored as in panel B. Underrepresented IS families are colored in gray to simplify the visualization.

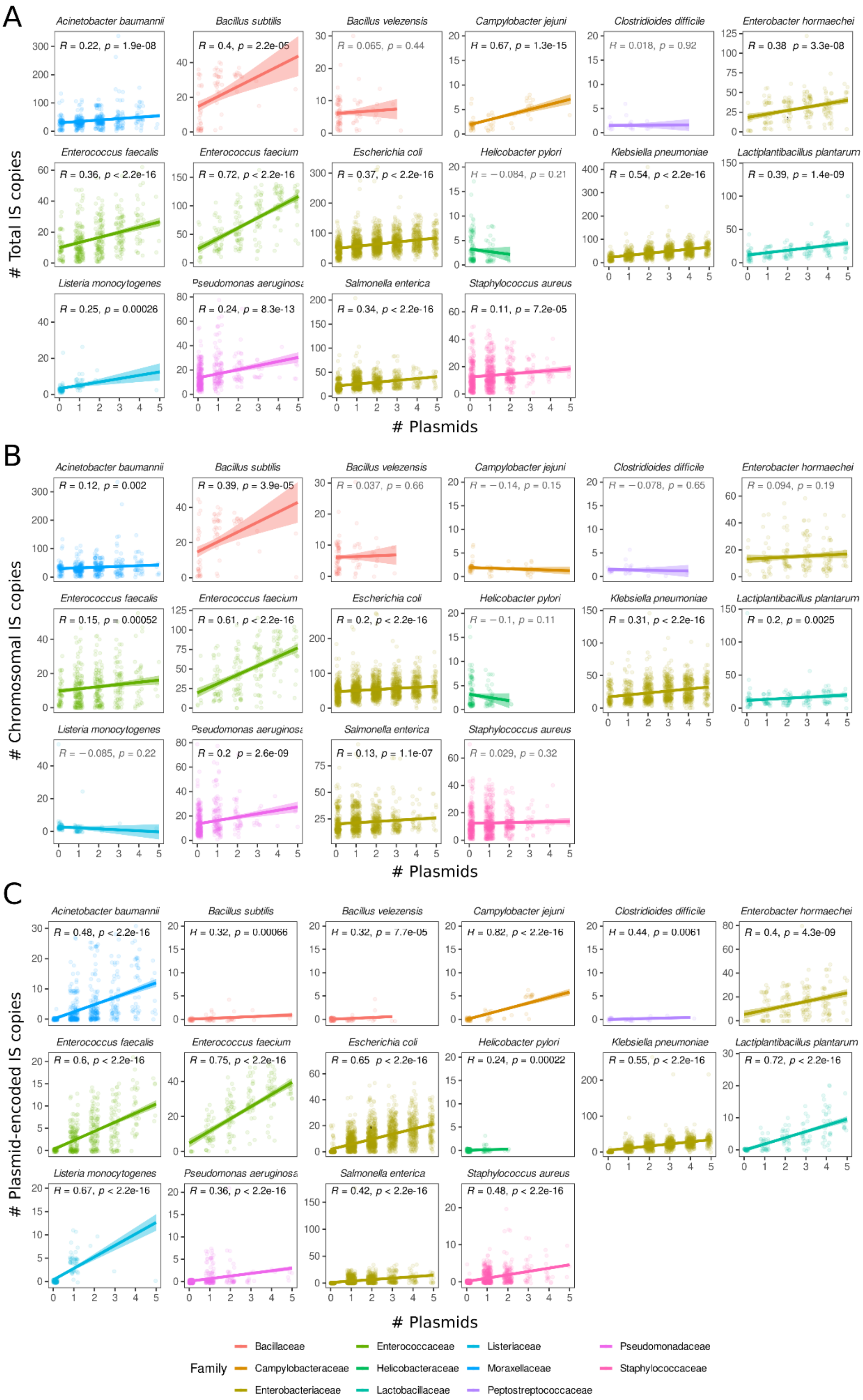

Supplementary Figure 3.- Correlation between IS genomic copies and number of plasmids in BV-BRC genomes analyzed. Correlation between the number of total genomic IS copies (**A**), chromosomal IS copies (**B**) or plasmid-encoded IS copies (**C**) per strain (y-axis) and the number of plasmids they carry (x-axis) in the most prevalent species from the BV-BRC genomes analyzed. Pearson correlation R and p-values are shown for each species and correlation. Correlation statistical values are shown in black if the association resulted significant, or in grey if it was non-significant. Dots, regression lines and error area colours correspond to the bacterial family of each species.

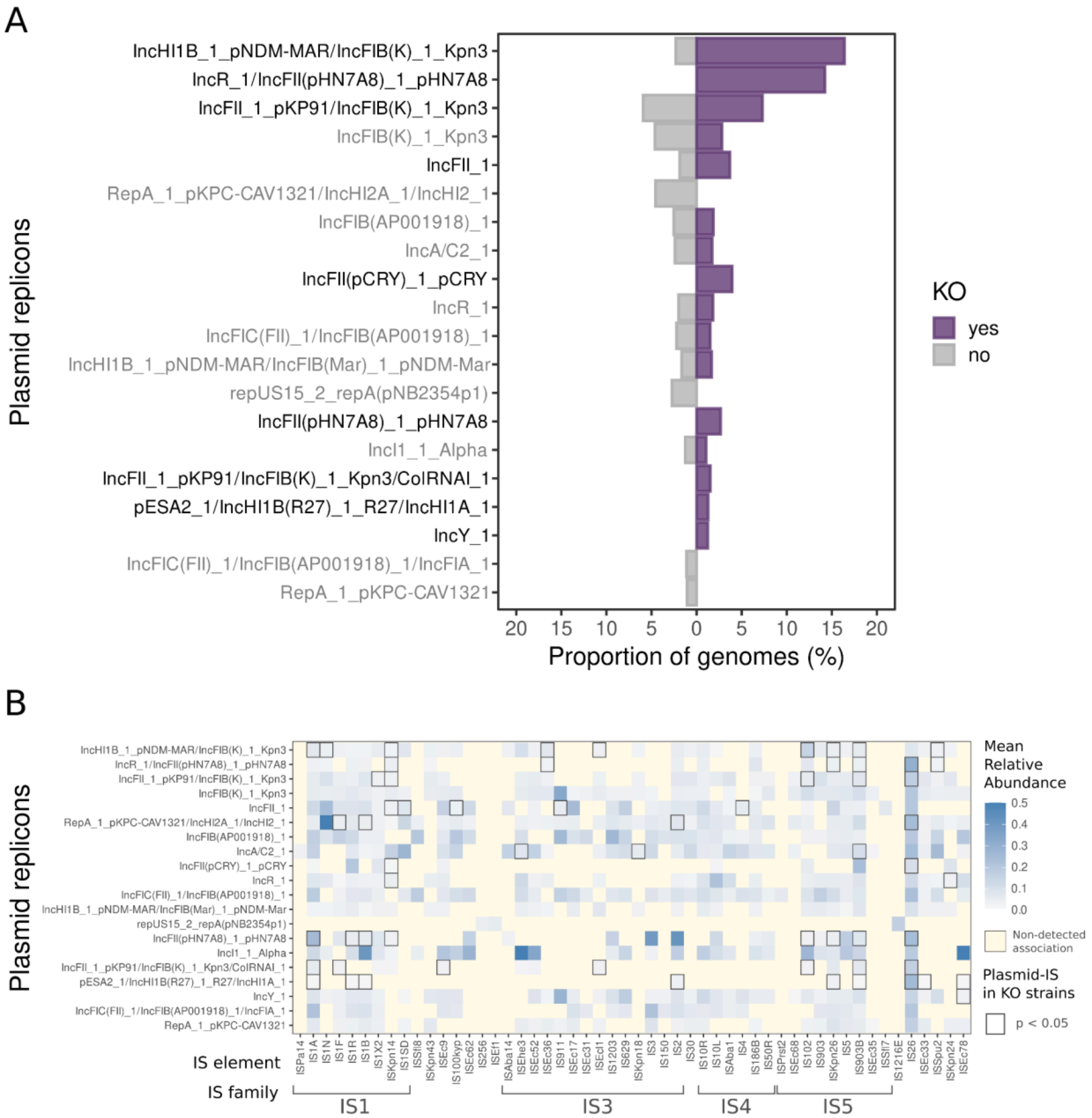

Supplementary Figure 4.- Statistical analyses of plasmid-encoded IS causing KO-AMR. A) Frequency of genomes from the BV-BRC encoding a determined plasmid replicon or fusion of replicons in strains with KO-AMR genes inactivated by IS elements (purple) or not (grey). The x axis indicates the frequency relative to the group not showing KOs (left, grey), and showing KOs (right, purple). The replicon names indicated with darker names were found significantly over enriched in strains showing KO-AMR inactivations by ISs (purple bars). **B)** Heatmap showing the abundance of plasmid-encoded IS elements. Y-axis indicates the most prevalent plasmid replicons. X-axis shows the IS elements driving the inactivation of the screened KO-AMR genes, sorted by their IS family in alphanumeric order. Only the top families causing KOs are highlighted. Each cell indicates the mean relative abundance (normalized per strain genome) of a given IS element encoded in a plasmid (i.e. if the mean relative abundance value of a Y-plasmid + X-IS combination is 0.1, it indicates that, if present, Y-plasmid encodes, by average, 10% of the X-IS element in a genome). Yellow cells correspond to non-detected associations. Highlighted cells with darker stroke lines correspond to plasmid-IS combinations significantly overrepresented (two-sided Fisher's exact test after FDR adjustment, $p < 0.05$) in the subset of strains showing IS-mediated KOs of KO-AMR genes.

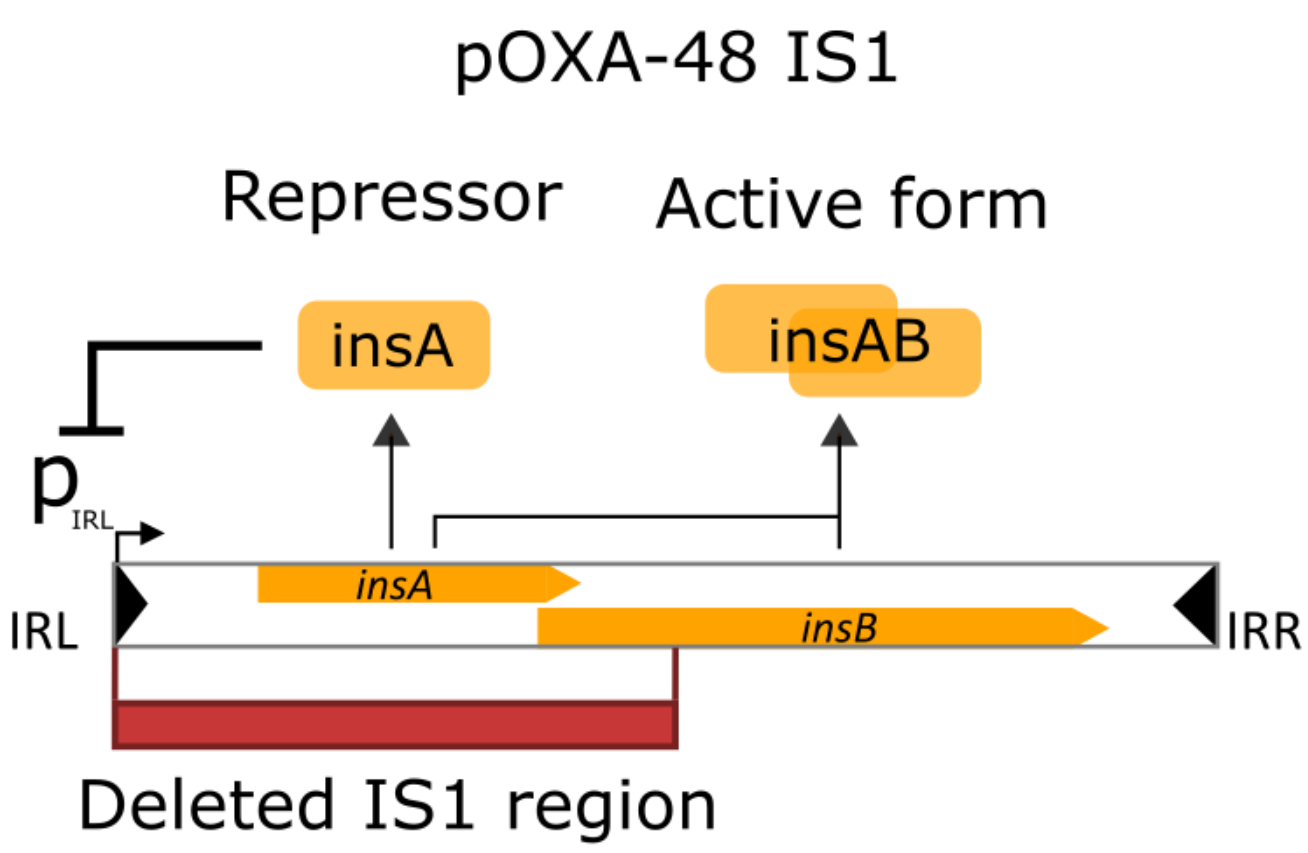

Supplementary Figure 5.- Schematic representation of a generic IS1 element copy and deleted region in negative control. The two open reading frames of IS1 (insA and insB) are represented in orange as arrows. Both resulting proteins (the active transposase InsAB and the repressor InsA) are also represented in orange above the ORFs from which they originate indicated by arrows. The black triangles in the ends of the IS1 are the terminal inverted repeats (IR) both left (IRL, containing the IS1 promoter pIRL) and right (IRR). The red bar represents the deleted region from the IS1 copies in pOXA-48ΔΔIS1. We deleted the promoter region as well as the repressor and part of the second ORF, avoiding both production of repressor, active transposase and interference of IRL with InsA produced by other copies.
